# Devil in the Dedifferentiated Details: A Rare Case of Primary Pulmonary Dedifferentiated Liposarcoma

**DOI:** 10.7759/cureus.66540

**Published:** 2024-08-09

**Authors:** Veronica Williams, Lewjain Sakr, Pablo Bejarano

**Affiliations:** 1 Pulmonary and Critical Care Medicine, Cleveland Clinic Florida, Weston, USA; 2 Pathology, Cleveland Clinic Florida, Weston, USA

**Keywords:** lung cancer surgery, sarcomatoid cancer lung, primary pulmonary leiomyosarcoma, primary pulmonary liposarcoma with leiomyosarcomatous differentiation, pulmonary liposarcoma, dedifferentiated liposarcoma

## Abstract

Primary liposarcoma of the lung is an exceedingly rare phenomenon, with fewer than ten cases reported in prior literature as of 2024. Rarer still, dedifferentiated liposarcoma of pulmonary origin is even less frequently reported, with only two cases having been described. Here, we detail the case of a 66-year-old female who presented with a large left-sided obstructing lung mass and underwent bronchoscopy with tumor cryoprobe debulking. Histological examination revealed a spindle cell sarcoma corresponding to a dedifferentiated liposarcoma with leiomyosarcomatous features as murine double minute 2 translocation was identified by fluorescence in situ hybridization and desmin by immunohistochemistry. The patient completed multiple radiotherapy sessions followed by pneumonectomy and a relatively uncomplicated postoperative course. As pulmonary liposarcoma tends to be poorly responsive to typical chemotherapy, standard management of pulmonary liposarcoma in the absence of metastases is surgical intervention to improve patient survival. Thus, we maintain that pulmonologists, oncologists, and radiologists should have a heightened awareness of this unique pathology in patients presenting with solitary lung mass.

## Introduction

Primary pulmonary liposarcoma remains an exceptionally rare pathological phenomenon, with primary pulmonary liposarcoma with dedifferentiation being even more uncommon. Dedifferentiated liposarcoma (DDL or DDLPS) is typically a non-lipogenic sarcoma that arises in the background of a well-differentiated liposarcoma (WDL), with dedifferentiation occurring in a small fraction of these cases [1]. Dedifferentiation refers to the emergence of cells with a loss of phenotypic specialization from an otherwise low-grade tumor, exhibiting a type of plasticity akin to that of early embryonic development [2]. DDL most often arises from the retroperitoneum or proximal extremities, and the lung is one of the most frequent sites of metastases [3]. Tumor dedifferentiation is known to be associated with increased tumor cell invasiveness and drug resistance; as such, primary pulmonary liposarcoma with dedifferentiation tends to be poorly responsive to typical chemotherapy, and surgical resection is recommended in the appropriate candidate.

## Case presentation

A 66-year-old female with a past medical history of thyroid Hurthle cell neoplasm, menorrhagia, and uterine leiomyomata status post-hysterectomy 10 years prior without histological evidence of malignancy presented to the hospital with a 10-month duration of progressive dyspnea, nonproductive cough, and, more recently, thoracic pain with deep inspiration. She denied any tobacco use history or recent sick contacts. She had previously been in good health and reported normal preventative cancer screenings regarding mammography and colonoscopy.

Vital signs were within normal limits. Neurological, cardiovascular, and abdominal examinations were unremarkable. A respiratory examination demonstrated absent breath sounds over the left lower lung fields but were otherwise normal on the right. No wheezing was observed.

The hemogram showed a normal hemoglobin of 14.9 g/dL and platelets were 314 × 10^3^/μL. A complete metabolic panel was unremarkable. The N-terminal pro-b-type natriuretic peptide was 64 pg/mL. C-reactive protein was 3.3 mg/L (normal range: 0-10 mg/L). The erythrocyte sedimentation rate was mildly elevated to 30 mm/hour.

**Figure 1:**
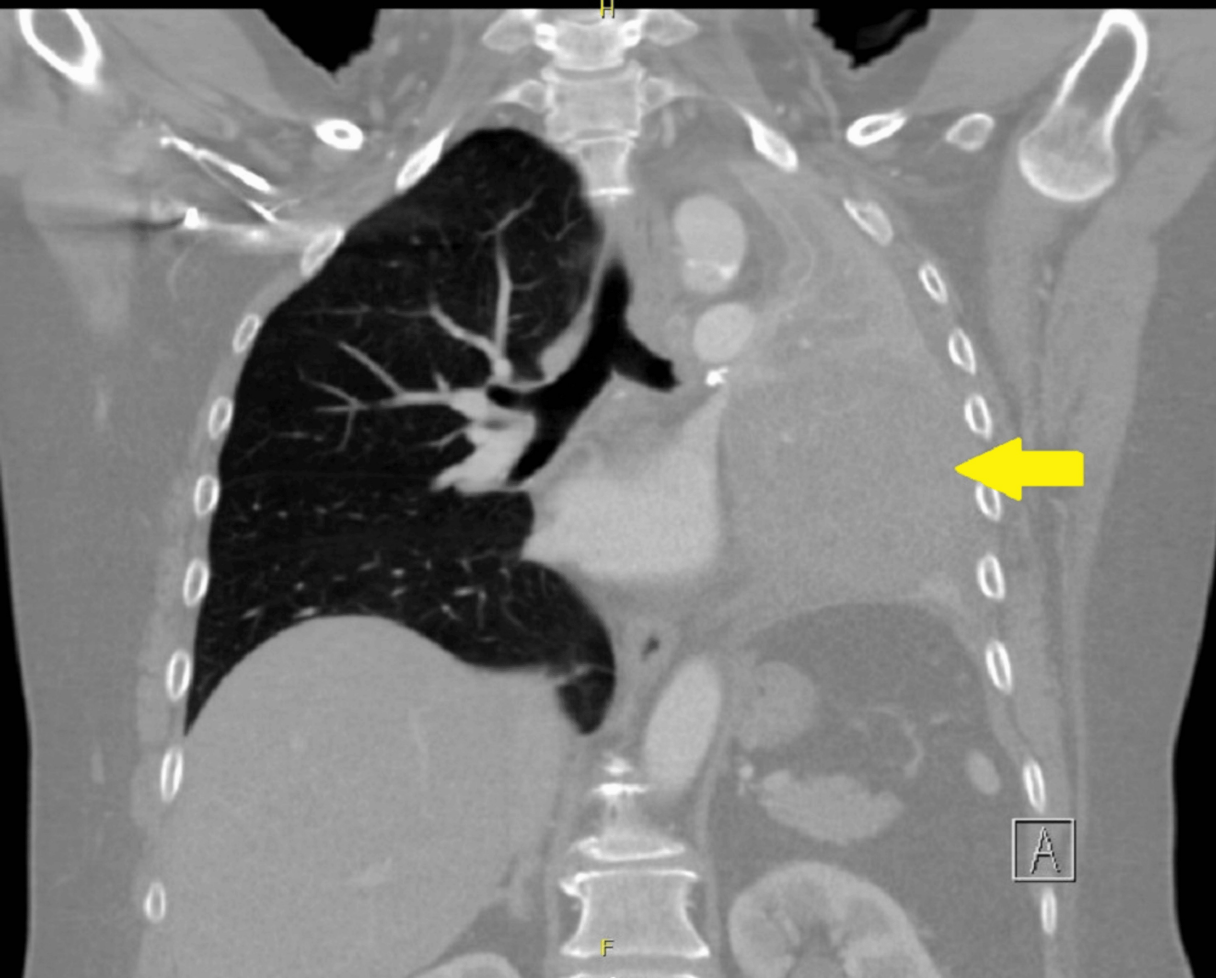
CT of the chest with heterogeneous mass-like consolidation in the left upper lobe (yellow arrow) and total occlusion of the left upper and lower lobe bronchi.

A CT scan of the chest showed a heterogeneous mass-like consolidation in the left upper lobe with left upper and lower lobe bronchi occlusion (Figure 1), prompting admission to the hospital for further evaluation.

**Figure 2:**
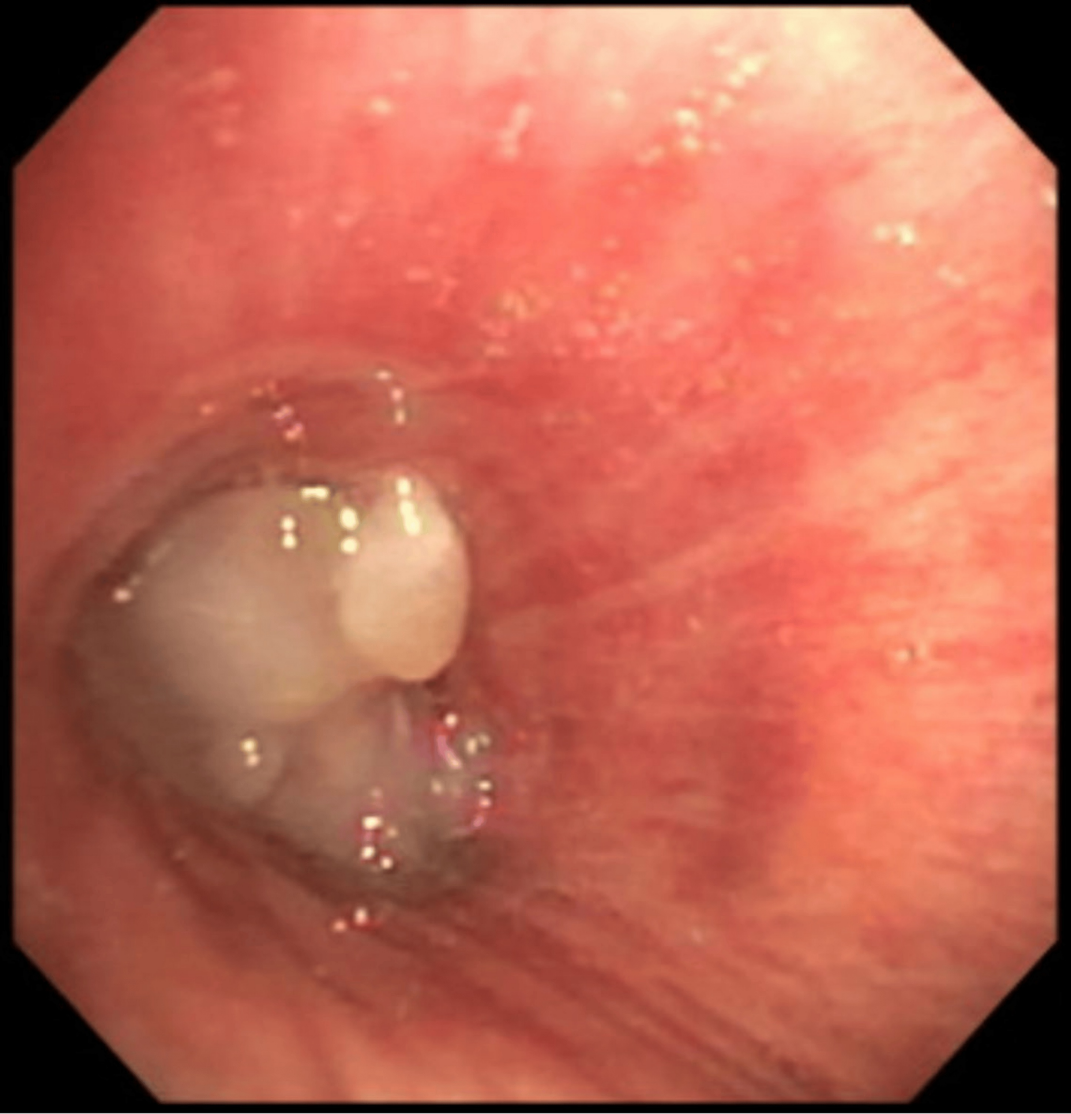
Distal left mainstem occlusion with completely obstructing pearly, white endobronchial tumor.

**Figure 3:**
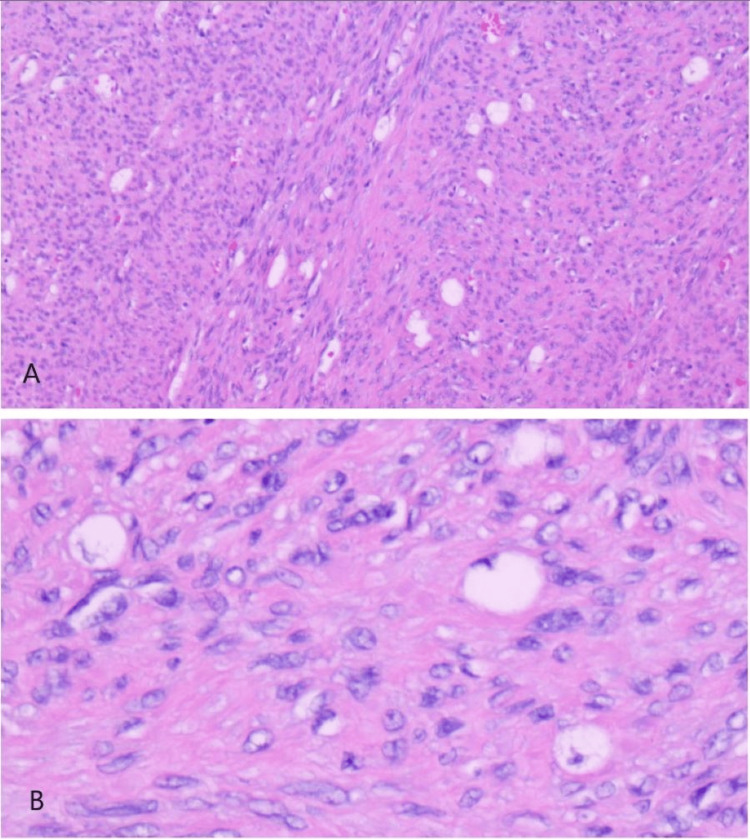
Histological examination of the resected mass. A: Interlacing fascicles of tumor cells (hematoxylin and eosin, 50×). B. Clear cells with scalloping nuclei (hematoxylin and eosin, 200×).

**Figure 4:**
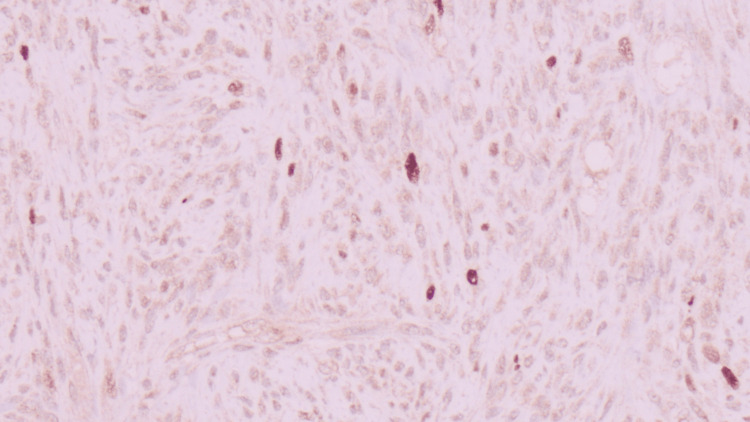
Immunohistochemistry staining of tissue positive for MDM2 iImmunoperoxidase; 110×).

She underwent bronchoscopy demonstrating a completely obstructing pearly, white endobronchial lesion within the distal portion of the left mainstem bronchus requiring cryoprobe debulking (Figure 2). Histological examination from biopsy and eventual resection was notable for residual lipoblasts (Figure 3), with positive fluorescence in situ hybridization (FISH) testing for *MDM2* gene amplification and positive MDM2 immunohistochemistry (IHC) staining (Figure 4). The tumor also stained for desmin. Stains for epithelial markers (cytokeratins) were negative. The final pathology demonstrated spindle cell sarcoma corresponding to a dedifferentiated liposarcoma with leiomyosarcomatous differentiation.

A follow-up CT of the abdomen and pelvis showed no signs of acute intra-abdominal process or mass. A subsequent positron emission tomography (PET) scan was initially concerning for involvement of hilar lymph nodes, and the recommendation was made for neoadjuvant therapy after a review of the case with the multidisciplinary sarcoma board.

**Figure 5:**
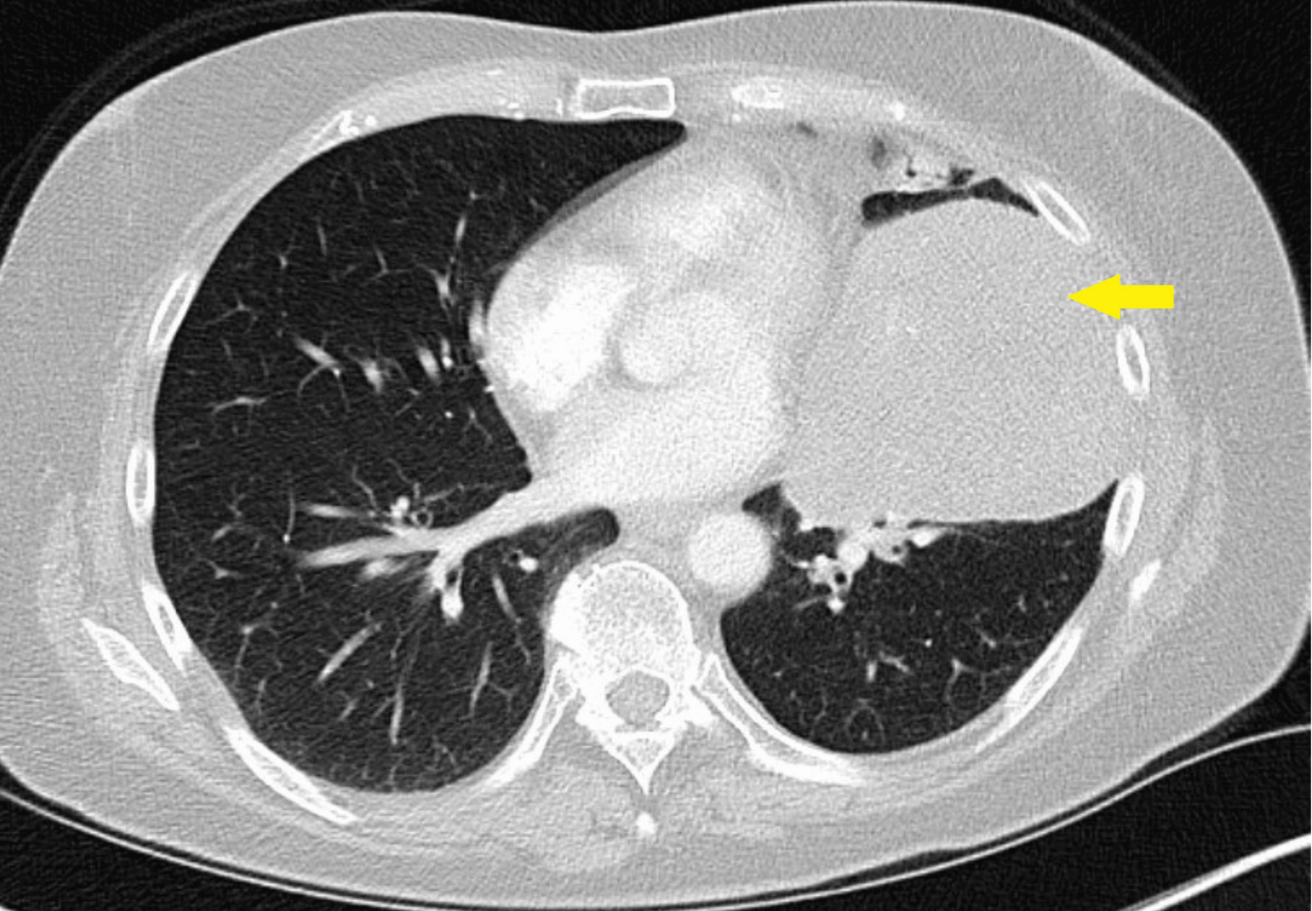
Reduction in the size of the mass (yellow arrow) post-radiation.

**Figure 6:**
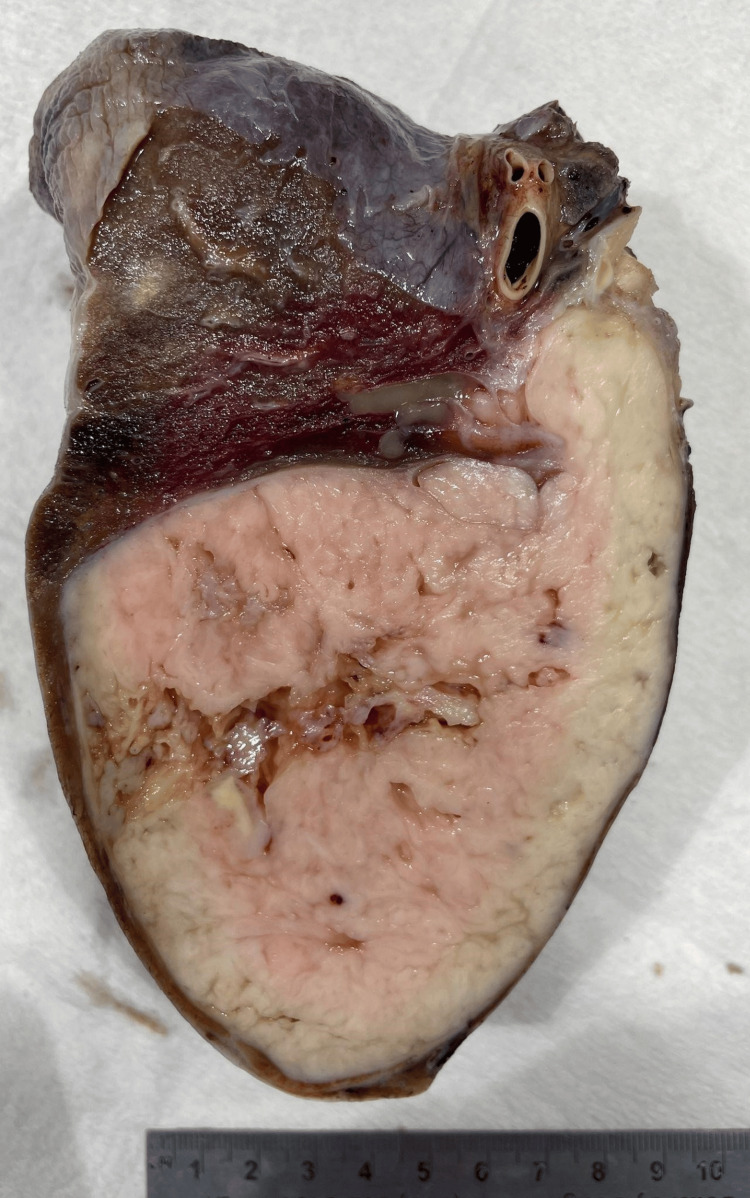
Left pneumonectomy showing the mass with a tan and hemorrhagic appearance in areas.

The patient elected for preoperative radiation resulting in an improvement in the size of the mass (Figure 5) with no further evidence of PET avidity in mediastinal nodes on the follow-up scan. Following the diagnosis, the patient underwent left-sided pneumonectomy (Figure 6).

A gross examination of the resected lung showed an encapsulated 13 cm mass with positive IHC for MDM2 and desmin. Margins were tumor-free with no metastases to lymph nodes or mediastinum. The patient had an uneventful postoperative course with no significant functional limitations thereafter.

## Discussion

DDL or DDLPS is typically a non-lipogenic sarcoma that arises in the background of a WLD, with dedifferentiation occurring in up to 10% of these cases. Tumor dedifferentiation refers to the emergence of cells with a loss of phenotypic specialization from an otherwise low-grade tumor, exhibiting a type of plasticity reminiscent of early embryonic development or regenerate processes. Tumor dedifferentiation is known to be associated with increased tumor cell invasiveness and drug resistance [4].

DDLs can exhibit a variety of heterologous lines of differentiation, including smooth muscular (leiomyosarcoma) and osseous types. DDL usually arises in the retroperitoneum or proximal extremities, with the lung being one of the most frequent sites of metastases. Primary liposarcoma of the lung is exceedingly rare, with fewer than 10 cases reported in prior literature [5-8]. To our knowledge, only two cases of primary pulmonary DDL have been previously described [9,10].

Cytogenetically, DDL is characterized by a supernumerary ring and giant marker chromosomes, both of which contain high-level amplifications of 12q13-15, including MDM2 and cyclin-dependent kinase 4 (CDK4) oncogenes. More recently, *CTDSP1/2-DNM3OS* fusion genes have also been identified within a subset of DDL [11,12].

*MDM2* is the main driver gene with the 12q amplicon, binding to p53 with negative regulation by preventing nuclear translocation and transcription and by promoting its degradation via an E3 ubiquitin ligase. CDK4 encodes a 33-kD protein that serves as a key factor in the regulation of the G1-S translation of the cell cycle. Subsequent accumulation of the CDK4-CCDN1 complex leads to phosphorylation of the tumor-suppressing retinoblastoma (RB) protein, triggering uncontrolled cell proliferation.

Our patient’s diagnosis of liposarcoma was based on the presence of translocation of the *MDM2* gene by FISH and IHC on the biopsy and the resected specimens, respectively. Additionally, the smooth muscle marker desmin stain was positive, confirming the presence of leiomyosarcomatous differentiation.

As a separate entity, primary pulmonary leiomyosarcoma is also extremely rare and can originate from the smooth muscle of the pulmonary parenchyma, pulmonary arteries, and bronchi [13]. In our case, however, the leiomyosarcoma arose from an existing liposarcoma; thus, we present this patient with pathological evidence of DDL with leiomyosarcomatous differentiation that originated from the lung as no evidence of extrapulmonary malignancy was found.

The standard management for localized DDL is surgery, with or without radiotherapy, as it exhibits a low response rate to conventional chemotherapeutic agents. In advanced disease not amenable to resection, first-line therapy is usually an anthracycline-based regimen, with either single-agent anthracycline or anthracycline in combination with the alkylating agent ifosfamide [12], as well as molecular-targeted agents.

## Conclusions

Primary pulmonary DDL with leiomyosarcomatous differentiation is an extremely rare type of lung cancer. It is challenging to distinguish distinct types of rare pulmonary tumors, often necessitating the identification of molecular translocations to diagnose them correctly. The present case emphasizes the possibility of this tumor arising de novo in the lung. Early pathological examination for prompt characterization of the pulmonary tumor is key, and surgical intervention when no evidence of metastasis is important to consider for patient survival.
